# Prediction of Fluid Responsiveness by Stroke Volume Variation in Children Undergoing Fontan Operation

**DOI:** 10.1155/2020/2595960

**Published:** 2020-11-07

**Authors:** Yun'an Song, Huiyan Hou, Jie Bai, Hongbin Gu

**Affiliations:** ^1^Department of Anesthesiology, Shanghai Children's Medical Center, Shanghai Jiao Tong University School of Medicine & National Children's Medical Center (Shanghai), 1678 Dongfang Road, Shanghai 200127, China; ^2^Pediatric Clinical Pharmacology Laboratory, Shanghai Children's Medical Center, Shanghai Jiao Tong University School of Medicine & National Children's Medical Center (Shanghai), Shanghai, China

## Abstract

**Background:**

Fontan operation is a palliative medical procedure performed on children with single-ventricle defects. As postoperative success of the procedure largely depends on the preload volume, it is necessary to maintain an appropriate pressure gradient between the systemic vein and the left atrium to ensure the effective volume of systemic circulation. However, there is a lack of effective indexes to evaluate fluid responsiveness in Fontan patients. Stroke volume variation (SVV) is a dynamic hemodynamic parameter based on cardiopulmonary interaction in mechanical ventilation. This study is aimed at validating the sensitivity and specificity of SVV and central venous pressure (CVP) in assessing the fluid responsiveness of Fontan patients.

**Method:**

Sixty-four children with single ventricle who underwent modified Fontan operation between May 2018 and January 2020 were included in this study. Patients were administered 10 ml·kg^−1^ albumin for fluid challenge within 10 min after cardiopulmonary bypass. Before and after fluid challenge, the invasive arterial pressure module was connected to MostCare™ equipment to collect the cardiac index (CI) and SVV dynamically in a time window of 30 s at a frequency of 1000 Hz. According to the range of CI change, patients with ΔCI ≥ 15% were classified into the responder (R) group and those with ΔCI < 15% into the nonresponder (NR) group. Using SVV and CVP as indicators, the receiver operating characteristic (ROC) curve of the patients was established, and the area under curve (AUC), diagnostic threshold, sensitivity, and specificity were calculated.

**Results:**

The SVV values were 16.28% (25th and 75th percentiles 14.17%-19.24%) and 13.68% (25th and 75th percentiles 12.90%-15.89%) before and after fluid challenge treatment in responders, respectively, and the values were 18.60 ± 1.83 mmHg before and 20.20 ± 2.39 mmHg for CVP after treatment. The AUC of SVV was 0.74 (95% confidence interval (CI) 0.54-0.94, *P* < 0.05), and the cutoff value was 16%, offering a sensitivity of 50% and a specificity of 91.7%. Meanwhile, the AUC of CVP was 0.70 (95% CI 0.50-0.92, *P* > 0.05), and the cutoff value was 19.5 mmHg, offering a sensitivity of 58% and a specificity of 76%.

**Conclusion:**

SVV exhibited a good predictive value for fluid responsiveness in pediatric Fontan patients. Appropriate fluid therapy according to SVV could improve the cardiac function of such patients. *Trial registration*. This study was registered in Chinese Clinical Trail Registry on Jan 26, 2018. Registration number is ChiCTR1800014654. Registry URL is http://www.chictr.org.cn/showproj.aspx?proj=25019. This observational prospective study was approved by the Local Ethics Committee of Shanghai Children's Medical Center affiliated to Shanghai Jiao Tong University (SCMCIRB-K2017035).

## 1. Introduction

Fontan operation is a palliative medical procedure normally performed on children with birth defects of the heart, in which, the systemic blood flow of the heart is attached directly to the pulmonary artery [1]. As there is a lack of the pumping function of the right atrium, the ideal systemic venous (pulmonary artery) pressure (SVP) should be maintained between 12 and 15 mmHg and left ventricular end-diastolic (LVED) pressure between 7 and 10 mm Hg after Fontan operation, to maintain the transpulmonary gradient (TPG) at a rational level of 2-8 mmHg. During anesthesia management, it is even more necessary to optimize intravascular hemodynamics, fluid volume, cardiac output, and tissue perfusion. Unnecessary expansion of the preload volume may lead to adverse results and may also increase mortality [2]. In addition, some studies have highlighted that assessing the responsiveness of fluid challenge may help distinguish between postoperative cardiogenic and pulmonary circulatory failure, which is instructive for the subsequent treatment in Fontan patients [3]. To the best of our knowledge, there has been less study reporting the assessment of fluid responsiveness following Fontan operation. Although the reliability of SVV in predicting fluid response in children with congenital heart disease such as ventricular septal defects has been well demonstrated [4–6], whether SVV can also be used in Fontan patients remains unclear. Compared with CVP, the method and meaning of SVV calculation are different, and therefore, it is unclear whether SVV is suitable for patients with single ventricular circulation. In addition, there is little simple and effective golden standard method for the prediction of fluid responsiveness in pediatric patients [7]. This study is aimed at determining whether SVV and CVP could accurately evaluate the fluid responsiveness of children with Fontan circulation and provide evidence-based clues for clinically reasonable fluid therapy in such patients.

## 2. Methods

### 2.1. Patients

This study was approved by the Medical Ethics Committee of Shanghai Children's Medical Center in Shanghai, China (SCMCIRB-K2017035) and certified by China clinical research registration (Registration No. ChiCTR1800014654). All parents or guardians of the patients voluntarily signed the written informed consent before operation.

The total sample size was calculated by MedCalc Statistical Software (Version 15.2.2; Ostend, Belgium). First, we considered that fluid responsiveness was predicted adequately if the SVV had an AUC > 0.70. We postulated that 60% of the patients would be fluid responsive. Hence, for a statistical power of 80% and *α* risk of 0.05, on the assumption that the ratio of case number in the responder (R) and nonresponder (NR) groups was 1 : 1, we estimated that the study would require at least 52 patients. Conservatively predicting a drop out of 20% of the cases, we screened a total of 64 patients.

**Figure 1:**
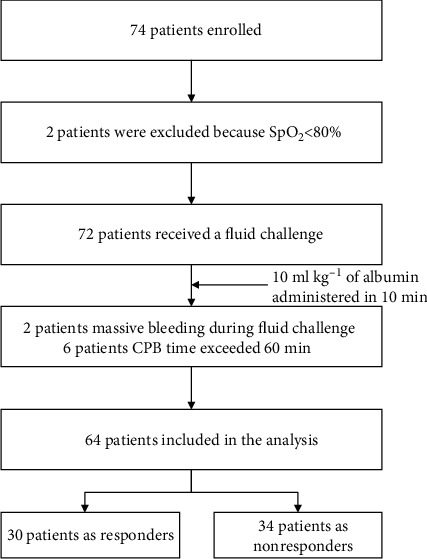
Flow chart.

This prospective single-center nonrandomized study included 74 children with single ventricle (American Society of Anesthesiologists (ASA) physical status II-III) who underwent modified Fontan operation under cardiopulmonary bypass (CPB) at Shanghai Children's Medical Center between May 2018 and January 2020. The inclusion criteria were children aged 3-8 years with ASA II or III who were scheduled for stage II modified Fontan operation with fenestration using CPB. The exclusion criteria were patients with arrhythmia before surgery with mean pulmonary artery pressure ≥ 18 mmHg, oxygen saturation lower than 80%, aged over 9 years, CPB time > 60 min, and the vasoactive–inotropic score (VIS) > 10 points [8]. Fluid challenge was observed in patients with sufficient hemostasis after CPB. The flow chart of this study is shown in Figure 1.

### 2.2. Anesthesia Management

All patients were premedicated with oral midazolam 0.5 mg·kg^−1^ 30 min before operation and then induced with intravenous (IV) midazolam 0.1 mg·kg^−1^, etomidate 0.3 mg·kg^−1^, sufentanil 2 *μ*g·kg^−1^, and rocuronium 0.6 mg·kg^−1^. Intubation was performed using a cuffed endotracheal tube. Mechanical ventilation was implemented by maintaining the pressure and keeping the tidal volume at 8–10 ml·kg^−1^, 4 cmH_2_O PEEP, fraction of inspired oxygen at 50%, the inspiratory to expiratory ratio at 1 : 2, and the respiratory rate at 14–20 times per min to maintain P_ET_CO_2_ at 30–35 mmHg. Anesthesia was maintained by using propofol 4 mg·kg^−1^·h^−1^, sufentanil 2.5 *μ*g·kg^−1^·h^−1^, and rocuronium 0.6 mg·kg^−1^·h^−1^. A 5.0 Fr double-lumen catheter was set up in the right internal jugular vein for monitoring CVP and positive inotropic drug administration. Mean value of CVP calculated during the entire respiration period was recorded. A 20G catheter was retained in the left femoral vein for fluid infusion. A 22G catheter was instrumented in the left radial artery to allow for routine arterial pressure and advanced hemodynamic monitoring by the pressure recording analytical method (PRAM) using the MostCare™ device.

Before cardiac resuscitation, dopamine 5 *μ*g·kg^−1^·min^−1^ was administered, and the dose of positive inotropic agents was adjusted by the end of CPB using the maximal slope of systolic upstroke (dp/dt_max_) monitored by PRAM and systolic arterial pressure (SBP). The dose of dopamine was reduced to 1 *μ*g·kg^−1^·min^−1^ in case dp/dt_max_ was >1.2 mmHg·ms^−1^ and SBP was >100 mmHg. Additionally, epinephrine was administered at 0.02–0.05 *μ*g·kg^−1^·min^−1^ in case dp/dt_max_ was <0.8 mmHg·ms^−1^ and SBP was <60 mmHg. Positive inotropic agents remained unchanged during the study period. Ten minutes after CPB and removal of the aortic cannulation, all patients received fluid challenge with 5% albumin 10 ml·kg^−1^.

### 2.3. PRAM and Hemodynamic Recording

The standard arterial pressure transducer was routinely connected to the monitor using an anesthesia workstation (Datex-Ohmeda Aisys CS^2^, GE Healthcare, USA), which was also directly connected to the MostCare™ to allow for transmission of the original signal and sampling at 1000 Hz [9]. SVV was calculated simultaneously as the variation of SV from the mean value during the most recent 30 s data and was displayed continuously using the following equation:

(1) $$\begin{array}{c} \text{SVV}\left(\%\right)=\frac{\left({\text{SV}}_{\max}‐\text{SVmin}\right)}{\left({\text{SV}}_{\max}+\text{SVmin}\right)/2}. \end{array}$$

Detailed 2 min measurements at 30 s intervals of each parameter were recorded by MostCare™ and then downloaded to Microsoft Excel for offline analysis. Subsequently, the four consecutive measurements were averaged and adopted before or after fluid challenge.

### 2.4. Study Protocol

This study was designed to evaluate the accuracy of SVV in predicting fluid responsiveness in pediatric patients undergoing Fontan operation. They received fluid challenge with 5% albumin at 10 ml·kg^−1^ for 10 min. Patients were defined as “responders” if CI increased more than 15% after a fluid infusion (group R), or “nonresponders” if the increase was less than 15% (groups NR) [10]. The medications remained unchanged during the study period. SVV was recorded by PRAM along with SBP, diastolic blood pressure (DBP), mean blood pressure (MBP), heart rate (HR), stroke volume index (SVI), systemic vascular resistance index (SVRI), CI, and CVP, before and after fluid challenge.

### 2.5. Statistical Analysis

All statistical analyses were performed using SPSS 20.0 (IBM Corp, Armonk, NY, USA). The Kolmogorov-Smirnov test was used to gauge the normal distribution of quantitative data, which are expressed here as means ± standard deviation (SD). Frequencies and proportions were used for categorical variables. Abnormally distributed data is represented by the median (25th and 75th percentiles) for continuous variables. Student's *t*-test or the Wilcoxon-test was used to evaluate group differences. The categorical data of intergroup were compared by Fisher's exact probability test. Variables of intragroup were compared before and after fluid challenge using the paired *t*-test or Friedman test. ROC curves were established to assess the capacity of SVV and CVP for predicting fluid responsiveness. The optimal cutoff was confirmed when the sum of sensitivity and specificity was maximal. A *P* value of less than 0.05 (2-sided significance testing) was considered statistically significant in all analyses.

## 3. Results

No significant adverse event occurred in any of the 64 Fontan patients during the study protocol. Among them, 30 were responders and the remaining 34 were nonresponders. The clinical data of all included patients are shown in Table 1. There was no significant difference in gender, age, height, or weight between the two groups (*P* > 0.05). All of the children undergoing correction surgery of the congenital heart disease received a continuous dose of dopamine (3-7.5 *μ*g·kg^−1^·min^−1^) before weaning from CPB; 6 children were additionally administered with a low-dose adrenaline infusion (0.02–0.05 *μ*g·kg^−1^·min^−1^). All VIS were <10 points.

The hemodynamic parameters before and after fluid challenge in groups R and NR are shown in Table 2. There were significant differences in hemodynamic parameters (SBP, DBP, MBP, SVV, CI, and CVP) before and after fluid challenge in group R. SVI increased insignificantly after fluid challenge in the R group (*P* = 0.19) company with the NR group (*P* = 0.21). Although the CVP of the two groups were relatively high and exceeded the reference value range of normal children, they were still in the normal range of Fontan patients.

**Figure 2:**
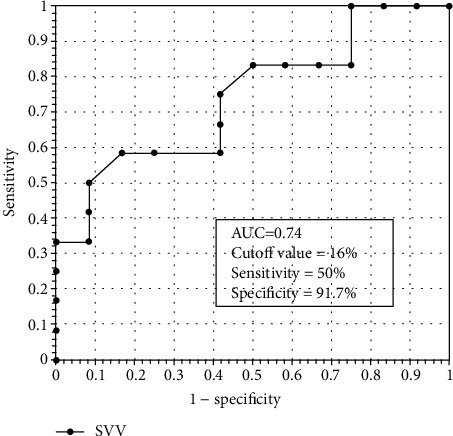
Receiver operating curves of SVV to predict fluid responsiveness in Fontan patients. AUC was 0.74 (95% CI 0.54-0.94, *P* < 0.05), and the cutoff value was 16%, offering a sensitivity of 50% and a specificity of 91.7%.

**Figure 3:**
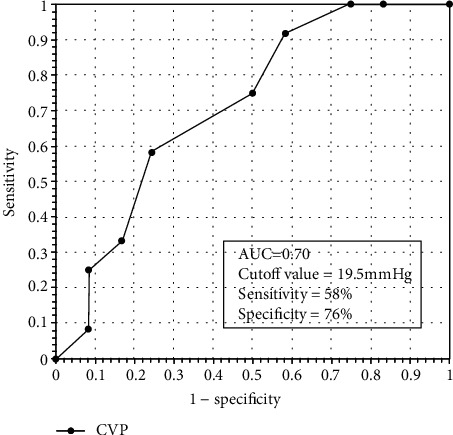
Receiver operating curves of CVP to predict fluid responsiveness in Fontan patients. AUC was 0.70 (95% CI 0.50–0.92, *P* > 0.05), and the cutoff value was 19.5 mmHg, offering a sensitivity of 58% and a specificity of 76%.

The AUC of SVV was 0.74 (95% CI 0.54-0.94, *P* < 0.05) and the cutoff value was 16%, offering a sensitivity of 50% and a specificity of 91.7% (Figure 2). The AUC of CVP was 0.70 (95% CI 0.50-0.92, *P* > 0.05) and the cutoff value was 19.5 mmHg, offering a sensitivity of 58% and a specificity of 76% (Figure 3).

SBP, DBP, MDP, SVV, CI, and CVP values were significantly different before and after fluid challenge in group R. SBP, DBP, MBP, and SVV values were significantly different between responders and nonresponders before fluid challenge. SVV was significantly different between responders and nonresponders after fluid challenge.

## 4. Discussion

PRAM is a method for monitoring continuous cardiac output based on changes in arterial pressure, which, in turn, is based on radial expansion caused by changes in the volume of a given blood vessel. Many studies have demonstrated a good correlation of PRAM with other classical methods, such as cardiac catheterization and Doppler ultrasound [11, 12]. SVV is a dynamic hemodynamic parameter that reflects changes in stroke volume [13–15]. SVV in patients with mechanical ventilation is less than 10-15%. According to cardiopulmonary interaction under mechanical ventilation and Frank-Starling principle, a change in stroke volume caused by mechanical ventilation is more significant when the blood volume is insufficient, showing a negative correlation between SVV and the blood volume [16, 17]. Theoretically, SVV can therefore be used to estimate the intravascular volume state and predict the responsiveness of the circulatory system to the infusion treatment.

SVV has been used in predicting fluid response in children receiving cardiac surgery. Some studies have shown that its accuracy is higher than CVP [18, 19]. To the best of our knowledge, this is the first study that demonstrated the good predictability of SVV for assessing fluid responsiveness after fluid challenge in pediatric Fontan patients, proving that it is a simple, fast, direct, and noninvasive method with good reproducibility. SVV can be measured with high-quality values in any individual patients. In pediatric anesthesia, some experts recommend 10-20 ml·kg^−1^ of fluid bolus for fluid challenge; there is no agreed formulation for a standard fluid load although almost all studies use approximately the same formulation; the types include packed red blood cells, human albumin solution, and fresh frozen plasma. A number of different thresholds were used for fluid responsiveness. The most common definition, change in stroke volume of >15% as measured by transesophageal or transthoracic echocardiography, seemed reasonable, as 15% is more than the expected error of measurement and is generally considered clinically relevant [20, 21]. In this study, we implemented aggressive fluid therapy by infusing 10 ml·kg^−1^ 5% albumin instead of crystalloid solution within 10 minutes. The result of our experiment demonstrated that the hemodynamic parameters including the MAP and CI were significantly improved in the Fontan patients after fluid challenge, as represented by a stable hemodynamic state, a balanced internal environment, an increase in urine volume, and the absence of significant adverse events. SVV < 16% indicated a sufficient blood volume in the patient, and continuous volume expansion treatment had little effect in further improving the cardiac function in such a condition. Therefore, we believe that an appropriate volume and fluid type are primarily important for improving the circulation capacity and cardiac function of Fontan patients after operation. This may be related to the increase in TPG and pulmonary forward blood flow, though it needs to be verified in future research.

The lung and blood vessel wall compliance in children is better than that in adults. However, the lung of children with a Fontan procedure is pathologically different from that of adults with a biventricular structure [22], which causes an increase in pulmonary vascular resistance and a decrease in pulmonary vascular compliance, and ultimately reduces the sensitivity of SVV. The higher positive airway pressure caused by mechanical ventilation will significantly reduce pulmonary blood flow and LVED volume. Because of the abnormal structure of the right atrium and right ventricle in Fontan patients, in which pulmonary vascular resistance (PVR) is relatively high, SVV produced by cardiopulmonary interaction may be significantly different between them and normal children. A high PEEP would increase RV afterload while reducing the systemic venous return and may depress the left ventricle (LV) dysfunction, which would result in the reduction of the predictive ability of the threshold value for SVV. To standardize the effects of mechanical ventilation in fluid responsiveness, tidal volume was set to 8-10 ml·kg^−1^ with a low PEEP (4 cmH_2_O). No significant difference was observed in lung dynamic compliance between the two groups, implying that the effects of lung compliance and mechanical ventilation to the experiment are similar [23–25].

There are a few studies reporting the application of SVV in children with single-ventricle Fontan circulation. In this study, we found that the cutoff of SVV was 16%, the sensitivity was 50%, and the specificity was 91.7%, suggesting that SVV is more specific than CVP in reflecting the volume state of children undergoing Fontan operation after CPB. Statistically, children with less SVV variation are more likely to be in a nonresponse state. Yoshitake et al. [26] used noninvasive hemodynamic monitoring to evaluate the cardiac output after Fontan operation by measuring the parameters of cardiac function in the patients with single left ventricular (SLV) and single right ventricular (SRV). Their results showed that the mean SVV (SLV : SRV) was 13.9% : 15.5%, which is similar to our study. However, the sensitivity and specificity of SVV in our study suggest that SVV may have a high false negative rate as a predictor of capacity reactivity in Fontan patients. Due to positive pressure ventilation, pulmonary vascular resistance may increase, causing a significant reduction of pulmonary blood flow in patients with single ventricle, which may lead to an increased SVV. This is one of the reasons for the higher false positive rate. In addition, the inhibition of myocardial contractility and the use of positive inotropic agents in early postoperative patients may affect the results. It is presumed that vasopressors would decrease SVV, whereas vasodilators would have the opposite effect. The hemodynamic effects of inotropic agents may have varying effects depending on their impact on LV ejection efficiency, vasomotor tone, and HR. Kim et al. [27] documented that inotropes do not alter SVV in an animal model. Mehrnaz et al. [28] demonstrated that vasodilator therapy increased SVV from 9% to 15%, whereas increasing inotropes or vasoconstrictors did not alter SVV. To reduce the impact of different doses of vascular drugs on the results, we did not adjust the dose of cardiovascular active drugs during data collection unless the patient's CI and dp/dt had significantly decreased.

We believe that SVV is more suitable as a fluid responsiveness parameter instead of volume expansion therapy for patients with low cardiac function. As each inspiratory and expiratory during mechanical ventilation can cause change in stroke volume, SVV can be used to indicate fluid responsiveness and the current volume state of the patient.

This study has some limitations. The fluid responsiveness of Fontan patients is affected by many factors; but as we only observed the effect of fluid challenge, the influence of mechanical ventilation on the research results could not be excluded. In addition, we observed the result of fluid response immediately after fluid challenge treatment without tracking changes in dynamic parameters in the cardiac intensive care unit. Finally, we did not use PiCCO as the control parameter for cost consideration.

## 5. Conclusion

In summary, SVV has a good value for predicting changes in fluid responsiveness in pediatric Fontan patients, although it may be affected by respiration and special physiological and anatomical structures. Appropriate fluid challenge therapy can significantly improve the hemodynamic of patients and increase the cardiac output, devoid of significant impact on the internal environment. We believe that SVV can be used as a monitoring indicator in carrying out appropriate fluid challenge treatment in pediatric Fontan patients, knowing that it can positively improve the cardiac function of the patients.

## Figures and Tables

**Table 1:** Clinical data of 64 Fontan patients in responders and nonresponders.

Parameter	Responders	Nonresponders	Total
Gender M/F (*n*)	30 (18/12)	34 (19/15)	64 (37/27)
Age (y)	4.97 ± 1.14	4.87 ± 1.27	4.85 ± 1.20
Height (cm)	98.76 ± 16.54	97.73 ± 17.43	98.00 ± 16.74
Weight (kg)	15.84 ± 5.69	15.64 ± 5.98	15.65 ± 5.37
Lung compliance (ml/cmH_2_O)	11.3 ± 4.2	13.4 ± 4.4	12.5 ± 4.4
VIS	7.1 ± 1.7	6.7 ± 1.6	6.9 ± 1.6

Data is shown as ($\bar{x}\pm \text{SD}$). There was no significant difference between the two groups.

**Table 2:** Hemodynamic parameters recorded before and after fluid challenge in responders vs. nonresponders undergoing Fontan operation.

Parameter	Before fluid challenge (BFC)	After fluid challenge (AFC)	*P* value of intragroup
HR (beats·min^−1^)			
Responders	117 ± 16.89	121.61 ± 15.93	0.12
Nonresponders	124.5 ± 13.64	122.73 ± 12.12	0.28
*P* intergroup	0.13	0.21	
SBP (mmHg)			
Responders	96.7 ± 12.32	102.4 ± 10.00^a^	0.031^a^
Nonresponders	95.71 ± 11.11	94.85 ± 13.83^b^	0.075
*P* intergroup	0.27	0.04^b^	
DBP (mmHg)			
Responders	48.40 ± 7.91	59.3 ± 7.25^a^	0.03^a^
Nonresponders	58.07 ± 7.80^b^	58 ± 10.67	0.38
*P* intergroup	0.04^b^	0.16	
MBP (mmHg)			
Responders	62.40 ± 8.23	75.3 ± 6.85^a^	0.02^a^
Nonresponders	72.07 ± 7.51^b^	70 ± 8.05	0.11
*P* intergroup	0.02^b^	0.05	
SVV (%)			
Responders	16.28 (14.17-19.24)	13.68 (12.90-15.89)^a^	0.02^a^
Nonresponders	15.19 (13.78-18.09)b	14.67 (12.88-15.89)^b^	0.07
*P* intergroup	0.03^b^	0.04^b^	
SVI (ml·m^−2^)			
Responders	33.06 (29.40-36.34)	37.98 (35.17-40.93)	0.19
Nonresponders	31.66 (28.54-37.63)	33.40 (29.57-36.97)	0.2
*P* intergroup	0.23	0.26	
CI (l·min^−1^·m^−2^)			
Responders	2.46 (1.89-3.21)	2.73 (2.19-3.42)	0.02^a^
Nonresponders	1.96 (1.32-2.45)	2.06 (1.64-2.70)	0.07
*P* intergroup	0.24	0.03^b^	
CVP (mmHg)			
Responders	18.60 ± 1.83	20.20 ± 2.39^a^	0.03^a^
Nonresponders	18.57 ± 2.34	19.64 ± 3.83	0.16
*P* intergroup	0.09	0.33	

Data is shown as ($\bar{x}\pm \text{SD}$) or median (25th and 75th percentiles). HR: heart rate; SBP: systolic blood pressure; DBP: diastolic blood pressure; MBP: mean blood pressure; SVV: stroke volume variation; SVI: stroke volume index; CI: cardiac index; CVP: central venous pressure; R: responders; NR: nonresponders. ^a^*P* < 0.05 compared with that before fluid challenge; ^b^*P* < 0.05 compared with responders.

## Data Availability

The datasets used and analyzed during the current study are available upon request.
